# Exploring parental perspectives of physiotherapy in children with congenital heart disease: a qualitative study

**DOI:** 10.1136/bmjpo-2025-003705

**Published:** 2025-11-10

**Authors:** Stephanie L Clarke, Julie C Menzies, Emma Shkurka, Nigel E Drury

**Affiliations:** 1 Department of Physiotherapy, Birmingham Children’s Hospital, Birmingham, UK; 2 Department of Nursing and Midwifery, School of Health Sciences, College of Medicine and Health, University of Birmingham, Birmingham, UK; 3 Paediatric Critical Care, Bristol Royal Hospital for Children, Bristol, UK; 4 Institute of Clinical Sciences, University of Birmingham College of Medical and Dental Sciences, Birmingham, UK; 5 Department of Physiotherapy, Great Ormond Street Hospital for Children, London, UK; 6 Department of Infection, Immunity & Inflammation, University College London, London, UK; 7 Department of Cardiovascular Sciences, School of Medical Sciences, College of Medicine and Health, University of Birmingham, Birmingham, UK; 8 Department of Paediatric Cardiac Surgery, Birmingham Children’s Hospital, Birmingham, UK

**Keywords:** Cardiac Surgery, Cardiology, Child Health

## Abstract

**Background:**

Children with congenital heart disease demonstrate developmental and functional impairment throughout their life. Published literature is limited but suggests postsurgical physiotherapy positively impacts developmental and functional outcomes in these children. We aimed to understand parental opinions of their child’s development or function, their experiences of physiotherapy services, how they feel physiotherapy could be used to support their child and what impacted access to services.

**Methods:**

A qualitative study using semistructured online interviews was conducted among parents of children with a diagnosis of congenital heart disease under the age of 16 years, who had undergone cardiac surgery in the UK. Data were collected between July and December 2024. The data were explored using reflexive thematic analysis and the study was reported in accordance with the Consolidated Criteria for Reporting Qualitative Research checklist.

**Results:**

12 semistructured interviews were completed involving 12 mothers and 1 father. Themes identified were parental priorities; understanding and experience of physiotherapy; access to physiotherapy and ideal physiotherapy service. Parental expectations of their child’s development were influenced by antenatal care and postnatal experiences. Parents continually evaluate the need for their child to access developmental and functional support. Engagement with physiotherapy is influenced by parental understanding, quality of service provision, impact of interventions on their child and the practical and emotional consequences to their family. Parents want physiotherapy services to deliver parental education and individualised support throughout their child’s life, particularly around key life events.

**Conclusions:**

Parental engagement with physiotherapy services is influenced by parental expectations of their child’s development and function, and their understanding of the potential benefits of physiotherapy. Physiotherapists should prioritise promoting and increasing awareness of physiotherapists’ role in children with congenital heart disease, alongside parental education and individualised care, to facilitate engagement.

**WHAT IS ALREADY KNOWN ON THIS TOPIC**

**WHAT THIS STUDY ADDS**

- We found variations in parental understanding and expectations of their child’s development and function. Parents described differing models of physiotherapy service delivery, which impacted their knowledge of physiotherapy. Priority areas for service provision included parental education and individualised physiotherapy interventions and services throughout a child’s life.

**HOW THIS STUDY MIGHT AFFECT RESEARCH, PRACTICE OR POLICY?**

- This work highlights the value parents put on physiotherapy services. Future research should aim to understand when parental education should be delivered and in what format. The impact of physiotherapy interventions, what format of care is optimal for improving outcomes of children and the feasibility of service delivery for families should also be examined.

## Introduction

 Children with congenital heart disease (CHD) are at risk of developmental delay. This has been attributed to a combination of abnormal in utero cerebral circulation, postnatal white matter injuries and further cerebral insults associated with surgical management,^1^ and can impact children across a variety of developmental areas including gross and fine motor control.^2^

Motor developmental delay can impact children throughout their formative years, with up to half of infants with CHD experiencing motor delay postcardiac surgery.^3^ In older children, this can manifest as impaired balance and coordination.^4^ Children with CHD physical limitations report a lower quality of life (QoL).^5^,^7^ Reducing the effects of CHD and improving the QoL of these children were identified as national priorities for research in the recent James Lind Alliance Priority Setting Partnership.^8^

Physiotherapy aims to improve or restore motor function and maximise movement ability. Multiple small studies demonstrate the value of physiotherapy for children with CHD, but heterogeneous populations and methods limit clinical application.^9 10^ Further research is needed to establish intervention effectiveness. Given the essential role of the parent in children with CHD, future research designs must recognise that family experience is crucial to their success.^11^ Currently, research exploring parental views on physiotherapy for children living with CHD is limited. A single study briefly explored caregivers’ views of physiotherapy in children aged 1–3 years. Parents reported physiotherapy to be beneficial and valued the involvement of siblings.^12^ However, there remains a paucity of evidence evaluating parental views on physiotherapy need and impact throughout a child’s life. Other studies have examined parental perspectives on developmental and rehabilitation services but did not specifically explore physiotherapy.^13^,^16^

Understanding parental perspectives on developmental concerns, current physiotherapy services and optimum intervention delivery will help clinicians provide care that aligns with the parents’ and child’s needs throughout their life. This knowledge can be used to support research designs, in addition to current physiotherapy service delivery. To explore in-depth parental perspectives, we chose a qualitative approach, allowing participants to share their experiences in their own words and facilitating an in-depth analysis and understanding.^17^ This study aimed to understand parental opinions of their child’s development or function, their experiences of physiotherapy services, how they feel physiotherapy could be used to support their child and what impacted access to services.

## Methods

### Design

A qualitative study using semistructured online interviews was conducted. The Consolidated Criteria for Reporting Qualitative research were used in the design and reporting of this study.^18^

### Patient and public involvement

The interview topic guide and recruitment strategy were shaped by feedback from six parents whose children had a diagnosis of CHD, and who are members of a local children’s heart charity *Young at Heart*. Three parents participated in a focus group reviewing the topic guide, one non-native English-speaking parent commented on the readability of the participant information sheet via a face-to-face meeting, and three parents offered asynchronous feedback on the recruitment strategy (see online supplemental file 1).

### Sampling

Parents of children with a diagnosis of CHD up to the age of 16 years, who had undergone cardiac surgery in the UK were approached, via social media, email and face-to-face using purposive sampling, to participate in this study. Parents whose level of English was not sufficient to fully engage in the interview process were excluded. The study aimed to recruit 15–20 participants, based on acknowledgement of CHD as a rare condition. With flexibility of the sample size in response to the quality and richness of the data collected.^19^

### Recruitment

Parents were recruited through two charities, *Little Hearts Matter* and *Young at Heart*, as well as a single National Health Service Trust. They were provided with a participant information sheet and given at least 24 hours to consider their participation in the research and ask questions before written informed consent was sought. Semistructured interviews were conducted by SLC, a female advanced physiotherapist undertaking a Masters in Clinical Health Research. The researcher had no established relationship with participants prior to the study, although she had had some contact with some parents as part of her clinical role.

### Data collection

A topic guide was compiled based on current literature (online supplemental file 2) alongside patient and public involvement and included four domains: (1) views on development and function; (2) experience of physiotherapy; (3) optimum physiotherapy service provision and (4) barriers to accessing care. Data were collected between July and December 2024. Interviews were digitally video and audio recorded via Zoom V.6.3.11 (Zoom Communications, San Jose, California, USA). Transcriptions of the interview were automatically generated as part of the recording process, pseudonymised and verified against the audio recording. Intelligent verbatim transcription was used in this study as it captured essential content while omitting filler words and repetition.^20^,^22^ Field notes were made following the interview to record key observations and initial interviewer’s insights. Risk to participants was deemed to be minimal but in the event of distress, participants were signposted to their local cardiac nurse specialist or Patient Advice Liaison Service.

### Data analysis

Data were analysed using reflexive thematic analysis (see online supplemental file 1).^23^ SLC independently coded the first three transcripts using MaxQDA (VERBI software, Berlin, Germany),^24^ prior to creating an initial coding framework. A second reviewer, JCM, an experienced qualitative researcher, used the coding framework to independently code two interview transcripts. The framework was then reviewed, minor amendments made and the remaining transcripts were independently analysed by SLC. The themes and subthemes were reviewed by the other coauthors (ES and NED) to clarify, amend and adjust themes until a consensus was reached.

## Results

**Figure 1:**
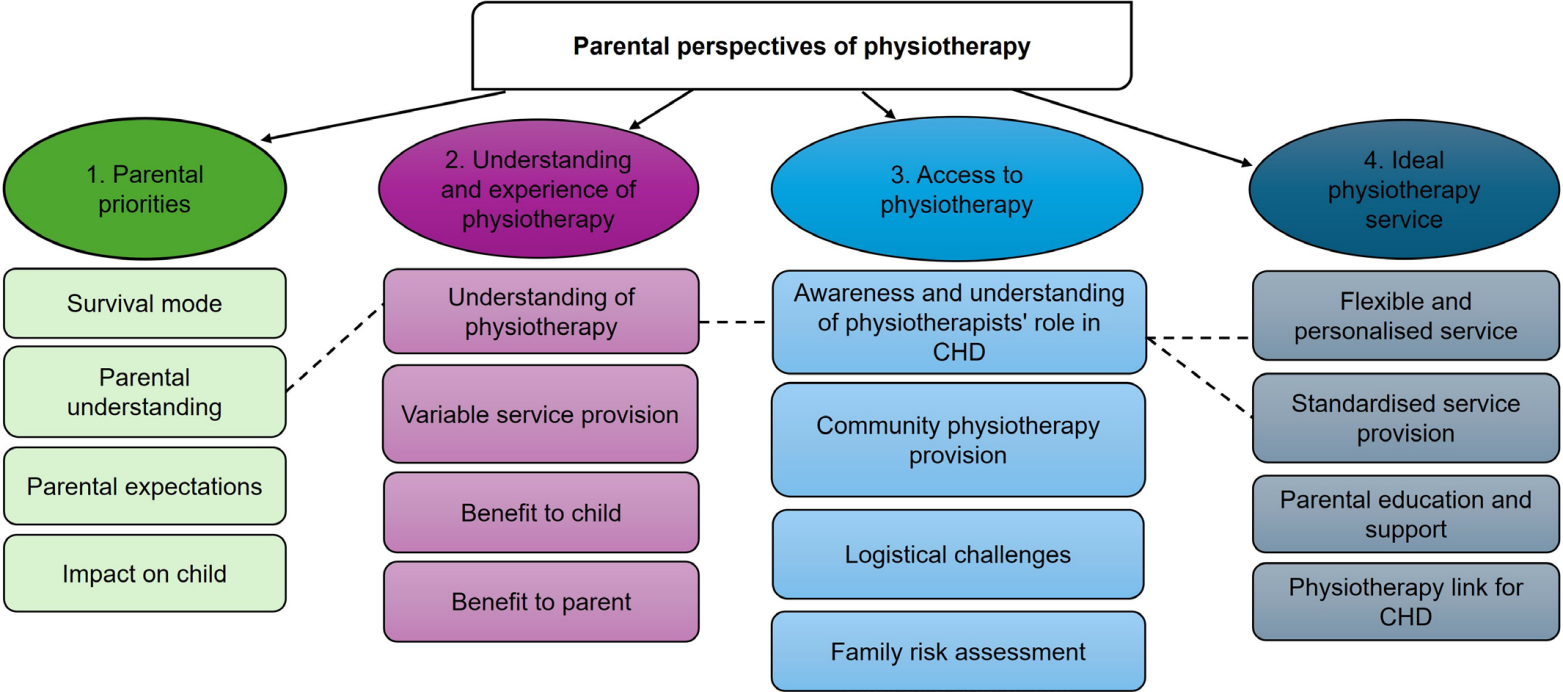
Parental perspectives of physiotherapy: themes and subthemes. CHD, congenital heart disease.

12 semistructured interviews were completed involving twelve mothers and one father, lasting between 30–90 min. Characteristics of the participants and their children are provided in table 1 and a summary of access to physiotherapy service provision in table 2. Non-participation data were recorded for the study (see online supplemental file 1). Themes and subthemes were developed (figure 1). The main themes were (1) parental priorities, (2) understanding and experience of physiotherapy, (3) access to physiotherapy and (4) ideal physiotherapy service. The results are described in detail below. See online supplemental file 1 for full list of data extracts used to support theme development.

**Table 1:** Participant and child demographic information

No.	Gender	Child’s age	Cardiac diagnosis*	Neurological diagnosis†
1	Mother	1.5 years	Truncus arteriosus, aortic stenosis, arrhythmia	None
2	Mother	7 years	Shones complex	ABI (bleed)
3	Mother	8 months	TOF, Left SVC	None
4	Mother	2 years	HLHS	ABI (stroke)
5	Mother	5 years	HRHS	None
6	Mother	5 years	HRHS	None
7	Mother	8 years	TOF	DiGeorge syndrome
8a	Mother	3 years	TGA	ABI (ischaemic injury)
8b	Father	3 years	TGA	ABI (ischaemic injury)
9	Mother	13 years	HRHS	ABI (arrest and bleed)
10	Mother	3 years	Tricuspid atresia, VSD, ASD	ABI (bleed), seizures
11	Mother	1 years	VSD, Tricuspid valve dysplasia, Pulmonary stenosis	None
12	Mother	2 years	HLHS	None

* Parental interpretation of cardiac diagnosis.

† Parental interpretation of neurological diagnosis.

VSD, ventricular septal defect.ABI, acquired brain injury; ASD, atrial septal defect; HLHS, hypoplastic left heart syndrome; HRHS, hypoplastic right heart syndrome; SVC, superior vena cava; TGA, transposition; TOF, tetralogy of Fallot.

**Table 2:** Parent reported service provision and parental concerns

No.	Inpatient respiratory	Inpatient development/rehabilitation	Community respiratory	Community development/rehabilitation	Concerned with development/ function	Concerned with fatigue
1	No	Yes	No	No	No	Yes
2	Yes	Yes	No	Yes	Yes	Yes
3	Yes	No	No	No	Yes	No
4	Yes	Yes	No	Yes	Yes	Yes
5	Yes	No	No	Yes	Yes	Yes
6	Yes	No	No	Yes	Yes	Yes
7	Yes	No	No	Yes	Yes	Yes
8a	Yes	Yes	No	Yes	No	No
8b	Yes	Yes	No	Yes	No	No
9	Unknown	Unknown	No	Yes	Yes	Yes
10	Yes	Yes	No	Yes	Yes	No
11	Yes	No	No	No	Yes	No
12	No	No	No	No	No	Yes

### Parental priorities

When their child was born, several parents reported being in ‘survival mode’, prioritising medical stability and feeding in preparation for cardiac surgery. After surgery, they focused on their child’s survival, recalling life-threatening medical events and feeling overwhelmed. Consequently, parents did not prioritise conversations around their child’s developmental trajectory.

They’ve probably got other stuff on their plate at the time so you know whether he can do this or do that really isn't important when his chest’s open (P3)

With two parents (P3, P1) stating it was not the right time for them to take in new information.

I couldn't physically cope with the beeping. I'd close the curtain and just cry and cry. I don't think I was ever going to be able to take in any information (P1).

Following the immediate postoperative period, parental understanding of the risks of developmental delay impacted their priorities. Two parents whose child followed a routine postoperative course (P1, P12) reported that they thrived and did not consider there to be an issue.

She first started to roll, it was maybe two months. She started to crawl, just before she got to four months, and then she walked the day after her 1st birthday (P12)

Two parents (P5, P11), whose child initially progressed well, understood that their child was not achieving motor milestones, and three parents (P2, P7, P10) recognised that leg pains impacted their child functionally. This shift in parental understanding prompted them to seek advice from healthcare professionals.

So, my dad’s got a very narrow straight hallway, he couldn't make it to the bottom of that without smashing into all the walls like a ping pong ball. So, we asked the cardiologist to see if he could refer us to someone (P6)

Parents of children who experienced an acute neurological event or prolonged hospital stay appreciated the potential for medium-term or long-term disability (P2, P4, P8a/b, P9, P10). Parents acknowledged their child was developmentally delayed and adopted a guarded approach to developmental expectations. One parent (P4) reported struggling to prioritise functional development when their child remained unwell.

I could roll him over, turn him, but there was so little we could do because most of the time he wasn’t well enough (P4)

Once discharged from hospital, thus during a period of medical stability, parents understood the need to prioritise motor development and reported relief when their child exceeded their expectations.

The 1st day she actually sat up we just cried knowing that she was making it (P8a)

Regardless of their journey, parental expectations of how CHD will impact their child were influenced by healthcare professionals. Antenatally, three parents (P1, P3, P7) were informed that their child may be physically disabled due to genetic conditions linked to the cardiac diagnosis.

I was told that he wouldn't walk, he probably wouldn’t talk (P1)

Parents were also prepared for the effects of fatigue. Consequently, four parents (P1, P4, P5, P7) acknowledged that fatigue influenced their child’s ability to keep up with their peers, leading to shyness and social isolation.

I think his tiredness holds him back. He started getting a lot shyer around children (P1)

Four parents (P2, P3, P5, P8a) noted that cardiac surgery, particularly having a sternotomy, impacted their child physically.

She’s had her chest cracked open like twice that’s got to do some damage (P8)

Parents described the impact of CHD on their child that they were not always prepared for. Three parents (P2, P7, P10) reported that leg pains caused their child distress.

If it’s an active day at school he can be up four to five times in the night with pain. Sometimes he’s crying with the pain (P7)

Parents expressed that their child developed an understanding of how CHD impacted them physically, with one parent describing her child’s awareness that she was different.

She’s obviously at that age now where she realises that the other people are doing it and that she can't do it, or that she wouldn't be very good at it (P2)

Parents of younger children (P3, P8a) stated their child had fewer opportunities to engage with age-appropriate activities due to the impact of living with CHD.

We’re a very outdoorsy family; we just couldn’t do those things with a child with an NG tube (P3)

Although parents understood that CHD and cardiac surgery could impact their child physically, they did not prioritise this in the short term. Parents accepted some physical symptoms were a consequence of their child living with CHD and adapted their lives accordingly. Long-term, when their child’s overall QoL was affected, parents prioritised accessing support.

### Understanding and experience of physiotherapy

All parents reported limited prior understanding of physiotherapy and how it could support their child. Many parents’ first encounter with physiotherapy was postcardiac surgery, when respiratory physiotherapy interventions were delivered. Four parents (P2, P3, P6, P11) reported finding this distressing even if it improved the child clinically.

When I first saw this physio come in and they’re banging her on the back, I was thinking, what are you doing to my child? I found it quite traumatic (P2)

Parents described a variable service provision that was influenced by the child’s journey. Parents reported that inpatient physiotherapists did not always introduce themselves or explain their role. This led to a lack of understanding of who had referred their child to physiotherapy, and why respiratory and developmental interventions were being delivered (P1, P3).

So, I’m assuming the nurse made the referral. Someone just came up to me and started moving him (P1)

For children with a routine postoperative course, six parents described no inpatient developmental or rehabilitative physiotherapy. Referral to community physiotherapy on discharge either did not occur or was facilitated once parents highlighted concerns (P2, P6, P7, P9).

Intense periods of inpatient physiotherapy were prompted by a new neurological diagnosis or prolonged length of stay (P2, P4, P8a/b, P9, P10).

When he had his strokes that’s then when we got a lot of physio intervention (P10)

These parents described access to community physiotherapy as an automatic referral process initiated at discharge. Parents characterised a robust community physiotherapy service provision for developmental interventions. Physiotherapy was primarily provided at home.

Multiple parents discussed the benefit physiotherapy had on their child. Of these, six parents (P2, P4, P5, P6, P8a, P9) noted their child completed developmental activities they initially thought were unattainable.

He can run up and jump on dad if he wants to. So, you know, that’s definitely physio that’s done all of that not just his normal development (P6)

Three parents (P2, P7, P10) highlighted the positive impact physiotherapy interventions had on leg pains and consequently their child’s ability to physically engage with their peers. Difficulties accessing referrals and a repetitive process of assessment and subsequent discharge led to parental frustration.

It’s just the discharge, re-referral, discharge, re-referral. It’s just frustrating (P9)

Parents also highlighted how physiotherapy services benefited them. Parents emphasised the positive experience of building a relationship with the physiotherapist (P2, P8a/b). They appreciated not having to continually repeat their child’s medical history as this was distressing for them and their child (P2, P9).

One big bugbear for me, each time you get referred to a new person, you have to go through a whole history, which can sometimes be quite triggering (P9)

Three parents (P4, P5, P8a) reported valuing how physiotherapists guided them in the best way to support their child using individualised exercises.

We were really supported by the people who did come and see us because he had his own personalised little exercise plan to do (P4)

These parents appreciated re-evaluation and progression of exercises which enhanced their motivation and provided reassurance of their delivery. With increased knowledge, four parents (P2, P4, P5, P6) reported feeling more empowered and able to justify the need for ongoing physiotherapy input.

I’ve got it built into her Education Healthcare Plan that she gets it done at school (P9)

### Access to physiotherapy

Awareness and understanding of the physiotherapist’s role in children with CHD impacted access to care. All parents described having limited initial understanding of why their child would need physiotherapy and any benefits. Parents who accessed minimal or no physiotherapy continued to demonstrate a reduced understanding. Consequently, three parents were unsure what services their child could access.

I wouldn’t even know what physiotherapy would be for her (P12)

Community physiotherapists lacked knowledge of CHD and did not always understand the impact of CHD on their child (P2, P7, P9). Parents lacked confidence in assessments and advice provided. They sought re-referral to services when they felt the physiotherapists did not address the underlying concern.

We did that, got discharged and then she started rolling her ankle a lot, went back to the GP, got referred again, got discharged again (P9)

Parents described the logistical challenges of accessing community and outpatient physiotherapy. Travelling long distances to hospital-based appointments took considerable time and impacted participation at work and school (P2, P5, P6, P10). This was compounded by many parents reporting that their child had multiple appointments with scheduling conflicts.

So next week the Thursday I have three appointments in three different hospitals come through for the same morning (P3)

Two parents (P6, P10) highlighted the value of having their own transportation due to varied public transport infrastructure. All parents acknowledged the financial impact of parking fees when attending hospital appointments but felt it was a necessary cost to ensure their child got the care they need.

The decision to access appointments was impacted by the emotional burden of engaging with physiotherapy within hospital settings. Parents described physical symptoms of anxiety and anguish when returning to the hospital (P2, P4, P6, P8a).

We’ve had some non-heart issues, she broke her arm, and I was like, I can’t go into the hospital*”* (P8a)

All parents reported that their hospital-associated anxiety was compounded by their child demonstrating significant distress when undergoing assessments by healthcare professionals.

The state he was in during his cardiac checkups you wouldn't notice anything physically anyway (P5)

Consequently, parents described feeling the need to risk assess accessing support. They evaluated the priority of physiotherapy assessment with the practical, physical and emotional consequence on themselves and their child.

### Ideal physiotherapy service

Parents considered it valuable for their child to access physiotherapy services throughout their life. However, they had differing views on optimal service delivery based on their experiences and understanding. Six parents (P1, P3, P4, P7, P9, P11) described a flexible and personalised service, including indications for physiotherapy, intervention frequency and location of service delivery to ensure it met the needs of their child and family.

Two parents (P1, P3) discussed the value of the cardiac nurse specialist having a central contact number or email and suggested a similar model could be used in physiotherapy services.

If you need to contact somebody, contact this person, give them an email (P3)

Parents also highlighted the benefits of having a physiotherapist linked to the cardiac service who had knowledge of CHD and the family’s journey, as they felt this would improve their confidence in services provided (P3, P5, P7, P8a, P8b)

I think it’s important to know that this child’s plumbing isn't the right way around. So, something might be a little bit more exhausting (P8a)

Six parents (P2, P3, P4, P5, P7, P9) appreciated the benefit of standardised service provision as a method of formal evaluation. Parents felt that it would be beneficial for physiotherapists to be present during cardiac clinic to ensure a multidisciplinary approach to their child’s care.

Like yearly assessments and reviews can be done 6 monthly (P7)

The frequency of formal evaluation varied between parents, although an assessment around key life events, especially presurgery and postsurgery, was highlighted by parents. Parents of older children were particularly keen for physiotherapy interventions postsurgery as they were concerned their child would lose functional skills.

I think going forwards the physio is going to be really crucial in his development, because if he spends a long time not being mobile again post-surgery, I'm worried he will lose some of those skills (P4)

Parents reported differing views on where physiotherapy should be provided. Some parents were willing to attend the hospital for one-off or infrequent appointments. Most families felt it was preferable for ongoing physiotherapy of greater intensity to be delivered in the community. The need for physiotherapists to reach into schools was considered important by three parents. All parents felt the main role of physiotherapists was to provide individualised parental education and support.

To know what to look out for, and things like that, if there are certain things you can do to improve (P11)

Two parents (P2, P3) suggested education around the physiotherapist’s role should be introduced during antenatal appointments.

It [physio] being included in the information would be really useful, I think, especially if it’s the parent’s 1st child or they’re unaware of, like myself, what physio actually did (P2)

Parents felt that understanding what physiotherapy could offer, including education and reassurance, would improve parental engagement with physiotherapy services.

## Discussion

This study provides unique insights into the perspectives of parents regarding their child’s development and function, their understanding of physiotherapy and consequently how services could be used. Parents described continually assessing and evaluating the impact of CHD on their child. Healthcare professionals’ narrative to families presurgery focuses primarily on survivability and risk of adverse events.^25^ Consequently, not all parents prioritise long-term outcomes.^16^ This is particularly true for parents who receive positive antenatal narratives that their child will live ‘a normal life’ postcardiac surgery.^26^ Parents do not anticipate negative functional outcomes as they have limited understanding that CHD can impact development.^27^ Introducing development as a new area of concern is not a priority for parents, and their ability to absorb information and prioritise physiotherapy to address this issue is low.^15 28^

Following the initial postsurgical period, variance in experiences and understanding impacts parental outlook regarding long-term outcomes.^12 13^ Some parents continue to have no concerns^25 26^ as they are happy their child is thriving.^16 29^ Other parents compared their child’s development to peers within a year of surgery,^12^ as they have had time to absorb new information provided.^15^ However, on average, parents of children living with CHD highlight concerns around 3 years of age.^14^ Unless education is provided, engagement with physiotherapy services will not be prioritised as parents may not perceive there to be a risk. Conversely, parents whose child has a prolonged hospital admission post-cardiac surgery develop increased knowledge of the need for physiotherapy.^16^ The understanding of physiotherapy interventions empowers parents to prioritise developmental input and provides security as short-term progress is tracked.^30^

When long-term outcomes are considered, parents place importance on their child’s QoL.^16^ Parents of children with chronic health conditions continually evaluate the need to access services, the perceived benefits/risks of engagement and the practical impact on their family.^31^ Differing models of service provision as described in our study reflect UK and Ireland physiotherapy services for children with CHD^32^ and provision for children with mild disabilities requiring surveillance.^33^ Parents face inconsistent service provision and a ‘watch and wait’ approach to community care, leaving parents unaware of available physiotherapy services.^26^ Parents are being asked to evaluate accessing physiotherapy support with limited knowledge of available services, a lack of knowledge of physiotherapists’ roles and challenges in accessing services.

Our study highlighted that most parents felt physiotherapy positively impacted their child and wanted input from services throughout their life. Early parental education is central to physiotherapy service provision, to ensure that parents recognise how CHD affects development and function at different stages and know to seek help.^12 16 26^ Structured evaluation allows early detection of developmental delay^25 26^ and provides a platform for parental education. Provision of parental education presurgery, and parental reassurance postsurgery, can lead to improved family experiences.^16^

It is important that physiotherapy services are flexible to meet the family’s needs^34 35^ and are centralised around one team to reduce parental administrative and emotional burdens.^13 29 36^ To improve parental understanding and family engagement with physiotherapy services, physiotherapists with knowledge of CHD should provide early parental education and a consistent individualised service.^37^

Limitations of this study include not reaching the prestudy recruitment target due to difficulties engaging parents in the immediate postsurgical period. It was concluded that additional participants would unlikely impact themes identified. Further limitations included a lack of paternal representation, with only one father participating. Greater representation from both parental roles would facilitate a more rounded analysis. Additionally, the study did not include non-native English-speaking parents, leading to underrepresentation of differing cultural views and experiences.

In conclusion, parental expectations of their child’s development are influenced by their understanding of the impact of CHD and interventions. Parents continually evaluate the need for their child to access services and the impact on their family. Physiotherapists should prioritise promoting and increasing awareness of physiotherapists’ role in children with CHD, alongside parental education and individualised care to facilitate engagement.

## Supplementary material

10.1136/bmjpo-2025-003705 online supplemental file 1

10.1136/bmjpo-2025-003705 online supplemental file 2

## Data Availability

All data relevant to the study are included in the article or uploaded as supplementary information.
