# Supplementary material for: Exploring parental perspectives of physiotherapy in children with congenital heart disease: a qualitative study
Source: BMJ Paediatr Open. 2025 Nov 10;9(1):e003705. doi: 10.1136/bmjpo-2025-003705 (PMC12603715; doi:10.1136/bmjpo-2025-003705)
Supplement: online supplemental file 1 [file bmjpo-9-1-s001.pdf]

SUPPLEMENTARY MATERIAL

Table S1: Summary of PPI activities and their impact

Area of PPI Input	Feedback	Changes made
Research question supported	The parents involved in the PPI process felt that this was an important area of research and many were keen for an intervention to be developed.	Nil
Research population	The parents agreed that it is important to include children who have undergone surgery as they feel these are the children who need the most help with their development. Parents advocated expanding the inclusion criteria from only parents whose child had accessed physiotherapy to any parents of a child with CHD. They felt that even though very few families had been offered physiotherapy post surgery, they agreed most parents would have an opinion on how their child could be supported to achieve developmental milestones, therefore, the study should have broader inclusion criteria.	Inclusion criteria expanded
Recruitment and Dissemination	The parents provided insight into the recruitment process. The parents all felt a recruitment poster would be useful for outpatient areas. They suggested the use of leaflets, so parents could take them home to think about after their appointments.<br><br>The parents all highlighted that they are keen to hear about anything that would help their child. Suggestions included a feedback event hosted by the charities and a generic summary distributed by email and over social media (not just social media as these are not always seen). The dissemination plan therefore includes a feedback event for participants and families associated with the charities involved in this study and dissemination of a plain English summary via the charity's newsletters which will go via email.	Included recruitment poster and leaflet<br><br>Dissemination plan expanded
Piloting and development of topic guide questions	The parents improved awareness of what topics are important and should be discussed within the interview. A set of pre-determined questions were piloted during the focus groups and questionnaire and feedback was gained from the parents. They all found answering questions about physiotherapy at the start of the interview challenging. All parents found the topic and questions regarding their child's development were important and should be included within the topic guide. Parents did not feel exploring the topic of "what is physiotherapy" was important. They felt it was more important to understand their experience of physiotherapy. Parents also felt the topic of how physiotherapy could help their child was important and was something they could explore even if they had not accessed physiotherapy previously. Parents did not feel a topic regarding guidelines was important to them and many expressed they did not know what a guideline was, therefore this was removed. The parents suggested alternative wording of questions to improve participant understanding. The areas included in the topic guide and question wording were amended to incorporate the feedback from the parents.	Amendments made to topics for discussion and wording of questions
Clarity of the PIS	The parents all felt the PIS was self-explanatory. They did not have any concerns with wording. This was also true for a parent who did not read English as her first language. No significant changes were made to the PIS.	Nil

Table S2: Literature to support topic guide development

Topic	Literature
Views on their child's development and function	Rogers et al 2023; Ware et al 2020; Ilardi et al 2020; Lisanti et al 2022
Experiences of physiotherapy	Ferrer-Sargues et al 2021; Fourdain et al, 2019; Hasbeba et al, 2017; Lamber et al 2017; Mitteregger et al 2024; Reynolds 2015; van Egmond- van Dam et al 2022; Cirovic et al 2014
Optimum physiotherapy service provision	Kaeslin et al 2023; Clarke et al 202; Bolduc et al 2022; Tikkanen et al 2023;
Barriers to accessing care	Abell et al, 2023; Abell et al 2024

Table S3: Six phases for reflexive thematic analysis

Phase	Description of process
1. Familiarise yourself with your data	Interviews transcriptions were verified against the recording. Reading of the whole data set by SC, allowed immersion in the data. Brief notes were made during this stage.
2. Coding	SC systematically reviewed the data to highlight key areas of interest, with a brief description. MaxQDA software was used to highlight data and record inductive codes.
3. Generating initial themes	SC and JM reviewed the coded data, and clusters of similar codes were combined which shared a common concept which might answer the research questions to form themes or sub-themes.
4. Developing and reviewing themes	The themes created were reviewed by NJ and JM and revised in relation to the code's extracts, the entire data set and research questions. A thematic map was created to review, alter, and refine the primary themes. Any discarded or new themes were recorded.
5. Refining and defining and naming themes	Ongoing analysis of themes and subtheme identified occurred, following by naming and describing each theme.
6. Writing up	Final analysis of the data using vivid data extracts in relation to the initial research question to produce a report of the analysis

Braun and Clarke, 2021

Table S4: Nonparticipation data at single NHS site

Date	Age child	Nonparticipation reason
23/8/24	6 months	Not interested in research
22/8/24	5 months	English not 1st language
22/8/24	9 months	English not 1st language
22/8/24	15 months	Basic English
22/8/24	13 months	Basic English
29/8/24	7 months	Not interested in study topic
29/8/24	6 months	Consent form signed- nil subsequent contact
30/8/24	2 years	Not interested in research
23/8/24	12 months	Not interested in research
29/8/24	3 months	Did not feel they were the right person for the study
5/9/24	1 month	Not interested in research
11/10/24	2 years	Consent form signed- nil subsequent contact
23/10/24	2 years	Consent form signed- nil subsequent contact
23/10/24	8 months	Basic English
28/10/24	6 weeks	Did not want to do an interview via Zoom
28/10/24	2 years	English not 1st language
28/10/24	16 months	Did not want to be recorded via Zoom due to religion
4/11/24	2 weeks	Not interested in study topic
6/11/24	1 years	English not 1st language

Full list of data extracts to support theme and subtheme development

Parental priorities

Survival mode

But I suppose [child] was quite poorly at the time. So it's hard isn't it, like it is a good time to catch a parent to ask, but also they've probably got other stuff on their plate at the time. So you know whether we can, you know, do this or do that really isn't important when their chests open (p3)

The beeping. I couldn't physically cope with the beeping. I'd close the curtain and just cry and cry and cry. I don't think I was ever going to be able to take in any information (P1).

the day before her surgery, the surgeon came up to us. She was so lovely, but she did say like, are you sure you want to go through with this surgery, and I said If I don't go through with the surgery, my baby's not going to survive (p12)

To be honest, it was all very much of a whirlwind horrendous time, so I don't remember much of It (p7)

I think it was more in the hospital, yeah, I think it was just that this is your given pathway of what would happen, which we would have grabbed with both hands. To be fair stuff, they could have said it, and we missed it in the craziness (p10)

She had a awful night, it was very touch and go whether she'd make it through the night (p8)

She was born 5 weeks premature as well so she had an hour and 40 min of internal CPR and then went on to ECMO for a week (p9)

Parental understanding

So my dad's got very straight, so we moved down here in with my dad during lockdown and he's got a very narrow straight hallway, which is only I don't know maybe 3 meters long, and he couldn't make it to the bottom of that without like smashing into all the walls like a ping pong ball the whole way down all that. You should probably get that looked at. So we asked the cardiologist to see if he could refer us to someone (p6)

I could like roll him over, turn him, sort of encourage him to turn and but again that was so little we could do, anyway, because most of the time he was in like he wasn't well enough (P4)

I don't want him to not develop you know. I was all the time doing stuff with him. Non, stop it was (P10)

he had his obviously chest cracked open so often because it was at 3 months and at 6 months if maybe so, he, you know, he hated tummy time kind of thing but also we couldn't

really do that kind of stuff that much, either so I don't know if that just meant his muscles developed a bit slower in those kind of respects (P5)

She's had her chest cracked open like twice that's got to do some damage (P8)

so he's quite and when you go and see him in the setting, you then see how far behind he is, because I think otherwise you kind of get used to the not even though (dad) and I both teachers we know developmentally where he should be but you kind of forget, don't you? Because that's just him at home. But when you see him in the setting or with peers, you're like, yeah he's massively behind (p10)

he was pretty much ready to turn over as we went into hospital, and then all of that went, and then, of course, like the turning, sitting, the beginning to crawl, basically just got put back. And I think if we hadn't have had the surgery at that point he probably would have been crawling slash sitting, you know (p3)

Quite a few months old I think we noticed he weren't able to do stuff (p11)

it relies on gravity to support with some of that stuff. So he needs to be up and about and yeah, walking developed enough to be able to get that (p4)

So he was pretty slow, I guess, at the beginning, very slow at the beginning, because it's obviously when you're 6 months old they had open heart surgery, and that means no tummy time, and all those physical things that you do to help them get physical I suppose (p6)

I think the fact that he'd got the CHD and then had his operation at 9 months. I think that that delayed a lot, because I think if he'd had his way he would have been walking quite soon, because he was he was starting to try and pull himself up at 9 months old, whereas he had the surgery at 9 months, so I think that slowed him down a bit (p9)

Parental expectations

I was told that he wouldn't walk. He probably wouldn't talk (p1)

It was early, when she 1st started to roll it was maybe 2 months, she started to crawl, she was just before she got to 4 months, and then she walked the day after her 1st birthday (p12)

the 1st day she actually sat up. We just I could have cried, you know, and knowing that she was making it (P8)

There's not really much wrong with him except from his heart (p1)

I think she's done far better than what was ever expected. So I'm always amazed by what she does actually manage (p9)

I would say, for an 8 month old, he's probably slightly behind where I perhaps would have put him but then, you know, you'd pass him in the street and go oh, he looks like happy, healthy baby, really like you wouldn't, you wouldn't know until you see his chest (p3)

I've been told that **** might not be able to participate in a lot of sports and things because he gets really tired, and I have noticed even taking him to a baby class (p1)

her PIC consultant asked them to come and see her originally, because she was making no movement whatsoever and they said by now that she should be making movements. She should be moving her arms and her legs, she wasn't doing any of that (p2)

we did 6 and a half months with a newborn, not a normal newborn and her life was very question mark on what it would be, how she would thrive, what, whether she'd need Physio, whether she'd need, what would she need? Cause no one could tell us (p8)

I was told during pregnancy that that was only a 60% chance that he will survive past 4 (p4)

So we did our 2 year check, and he hadn't got any of the things. None of them (p10)

so the health visitor said, so the health is really good to be fair and but she's like, Oh, I'm gonna come and do this 9 to 12 month check at 9 months and see what we're missing. I'm like he's not gonna meet most of those tick boxes. I know he's not right now, so I don't know if that's very constructive way of doing it, really (p3)

I was told from then the last 20 weeks of my pregnancy, that **** would potentially be a dying baby (p1)

we've had to fall really far behind. And we're still having to play catch up (p6)

Impact on child

I think his tiredness holds him back. He started getting a lot more shy around, children (p1)

when I was dropping her to school yesterday morning and her friends came running up her and were like [child], and then they got to her and then they ran away, and they were like, chase us [child] chase us and like literally, they were so far in front that she was never gonna chase them. She was never gonna catch up with them. But she tried, and then she stopped, and then she realised, you know what? I'm not gonna be able to do this. So she just turned around and walked back to me (P2)

it depends, if he has, so like for sports day at school. If he has sports day at school, it's absolutely horrendous on the night. So if it's an active day at school he can be up 4, 5 times in the night with pain. Sometimes he's crying with the pain, so it all depends on the level of activity during the week (P7)

She's obviously at that age now where she realizes that the other people are doing it and that she can't do it, or that she wouldn't be very good at it (P2)
You know all the things that you know we're a very outdoorsy family. We just couldn't do those things with a child with an NG tube, you know, in and out of hospital every 5 seconds for appointment here, appointment there (P3)

So she does really struggle with her leg pain. So I'd be in her room at like 2 o'clock in the morning because she's rolling around in pain because of her legs. So I'm doing stretches at like 2 o'clock in the morning, she's crying (P10)

I always know that on a Monday and a Wednesday guarantee she will be crying because her ankle is hurting her and it is repetitive (p2)

he will try and follow you around and follow his friends but I think he just, he can't in the same way keep up with them (p4)

I think his mobility issues has led to him, being quite nervous of his peers, because they're obviously so very fast. If we go to a soft play, then he'll want one of us to be there to be his legs because he can't play chase like the rest of them do, and it freaks him out when they run around playing chase (p6)

She's so far behind! I take her to like, try and take her to groups and things, and I feel really out of place because I'd just be like I don't know how to start, because I'm gonna cry (P8a)

Sometimes he is aware that he can't always keep up with them (p7)

He used to not really realize his limitations and now he's kind of aware enough of what's going on around him that he sort of knows he's starting to notice. He's different, and notice those limitations that he has (p4)

Other children particularly he used to be absolutely terrified of because they get in his face, which he didn't like, or so he couldn't get away from them. So it used to be that like a play group for another kid was within I don't know 2, 3 metres of him. He'd get really upset (p5)

I think his tiredness holds him back. He started getting a lot more shy around, children. He kind of lets children do whatever they want from him. So if a child snatches from him, he kind of just gives it to them (p1)

Understanding and experience of physiotherapy

Understanding physiotherapy services

They explained to me his legs weren't muscle ready, well, they weren't movement ready, or something, so where we can just get up and walk off, and we're all right, or we can just get up and run. He's not quite ready quick enough to be able to do that and just that he's got weak core muscles and shoulder muscles (p7)

So that was our 1st experience of physios was reinflating his lung (P6)

In hospital when he was a newborn, I had a physiotherapist come out, and it was to do with him moving (p1)

I know it's there to help, it’s your physical stuff, isn't it? So, it helps, like with any physical, like exercises and stuff that might be needed to help with the muscles and to get the joints moving, and things like that. As far as I'm aware (p7)

I see that it moves the secretions up and help the you know secretions come up better, and that actually she breathe a lot better (p2)

she's probably had physio input for respiratory and movement, and she had some neurodevelopment physio when she was small, probably for the past 6 years (p2)

we obviously we know the physio from the PICU, which is more to do with when she's intubated and she's having physical like physical Physio (p8b)

It can be a lot of different things, and everyone has a different role. But it's it's to aid the recovery of the child, and that could be neurologically, it could be respiratory, it could be developmentally that I mean, there's a whole host of aspects that it will help (p3)

When I first saw this Physio come in and they're banging on the back, I was thinking, what are you doing to my child? I found it quite traumatic. I was like, you're literally just smacking my child on the back (P2)

So I'm assuming the nurse made the referrals. Someone just came up to me and started moving him (P1)

I don't even know what was said in the kind of physios referral in terms of the referral from hospital to home (p4)

I think somebody came down when we was in intensive care. Towards the end of his week, in intensive care they came down and saw him first, But I don't know who asked for that (p7)

And you're just kind of in the middle trying to be like well, I do agree with what you're saying in principle, but also like he's on an oxygen tubing that's like 2 meters long so there’s not a lot I can do (p4)

I'm just grabbing the notes because I need to give [child] some treatment for his chest. You know, something like that doesn't just to say what they're doing, cause a lot of people would just like, Come, take some of his notes and then wander off again. I'll be like all right. Okay, what's going on there (p3)

The only thing I would say is, maybe at the start, when you do actually go in and start working with a family is to explain what you're doing and why (p10)

Variable service provision

The only Physio we've really had is like after surgery Physio like when they come and pat him on the chest and do suctions on him and stuff, but he hasn't had any other physio other than that (p11)

There was the Physio to help I think with the breathing, to clearing it off his chest (p7)

So he really only saw physio on PICU and that was everything from just coming to having a look and listen, up to treating (p3)

She's had Physio again as she got a bit older for movement, and she had community Physio (p2)

think community, like most of the NHS, they were extremely short staffed (p4)

And then, when he had his strokes that's then when we got a lot of physio intervention at [hospital] a lot (p10)

Because it just seems to be they, they see him, okay, you're discharged again. Now that's it (P7)

it's just the discharge re-referral discharge re-referral. Its just frustrating (p9)

one big bugbear of me and each time you get referred to a new person, you have to go through a whole history in front of her as well, which can sometimes be quite triggering and quite upsetting for her as well (p9)

We were under her for a year and but I think we had, really, we probably June. yeah, it's yeah it probably was a year, but I think towards the end of that year it wasn't every month. It was like, right, okay, I'll come see, I think we had like a 3 month break, because he had just progressed (p10)

They were coming every day, and then I think it went to like nearly every other day (p11)

like 3 times a week, then twice a week at the start (p2)

Benefit to child

she she's actually where she should be, which we from the child we brought home, and all the physio work we did (p8a

he can walk much better now than he used to (p6)

We had physiotherapy as a tiny baby to help her move. Sit up roll crawl, do all the positions that naturally babies would do (p2)

he gave us exercises at home, obviously which he really enjoyed. He's really like he's a very diligent kind of kid, so like he's got a job to he’ll do it (p5)

He can kind of run up there and jump on dad if he wants to. So you know, that's definitely Physio that's done all of that not just his normal development (p6)

You know they made they made it fun. You made it into a game for her (p2)

they would talk us through it, and they'd explain what they were doing and why they were doing it and show us how, we like to learn (p8a)

So you know, definitely did them every day and everything, and we were living with his granddad at the time and before we got our house and so he's got his granddad there on the floor, like, yeah be an egg now, grandad, and it's hilarious (p5)

So when she's doing it in less pain you know, she gets less pain and like I was saying with the hopping, you know, she's able to hop on one foot and got stronger (p9)

we were really supported by the people who did come and see us because he had his own sort of personalised little exercise plan to do (p4)

So I've got it built into her EHCP that she gets it [physio] done at school, because, you know, physio is like she needs to be doing this (p9)

Benefit to parents

It was primarily again to kind of give us some pointers on what we could do at home, to sort of take care of him, to give us some pointers on how we might be able to support him to do things, you know, in a more upright position (p4)

it's a big it’s a big thing for us at the moment she's actually caught up to all her peers, which we are in shock about (p8a)

we were very fortunate on in the hospital to have the same physios so we got to know them very well (p8a)

we hold physios really highly in our household and I worship physio’s a bit (p5)

That Physio is really good, because we have no idea what we're doing. I mean, if it wasn't for the physio even if she was born without any complications. We just kind of sit there and maybe YouTube, a video or something like how to make a baby walk (P8b)

I understand the common stuff and I've watched going the patty now, so I have an idea of. If he sounds chesty, I'll just pat his back, which he really likes me to do (p11)

we obviously had the physios who supported us to get her out of the ward when we could be a bit more mobile, which I just think I cried at them the 1st day we'd literally walked to the fishes on the on the ward, and I just burst like I was like, I can't believe we've just left the room. So we had the physios in that response as well (p8a)

You know I don't I can't recall ever having a bad experience physio-wise, and I always feel that whatever I, you know, if I had you know a question, or I wasn't quite sure I was thinking, you know, is this right? Everybody was quite approachable for me to ask you know ask, is this normal? And you would just say yes or no, or we can look at it, or just stop being silly, you know (p2)

I think it's a lot about as in in hospital, and you know you've got to get on with whoever you're working with, because you're trusting them with your child, and I think if someone

rocked up to me and said, Well, I know your child's this, that, and the other I'd suddenly go Okay, you know you know what you're in about, and I'd feel a bit more trusting (p8a)

I think they gave me enough at the time to kind of put my mind at rest that I wasn't going mad in terms of saying there is something wrong (p7)

Access to physiotherapy

Awareness of physiotherapy role in CHD

Before I had ****, I thought physiotherapists just sat in the office rolling on a gym ball. I didn't know what physiotherapy did, I didn't know what they were there for, and I just thought they were there, you know, if you got a bad back, basically (p2)

I said I think they've got the wrong bed number and they said, oh, we've just come to do physio and I said, isn't he a bit young to be doing exercise and they were like, oh, no, we're not. I'm really embarrassed to say this, because I was just like, Oh, my God, they're gonna be getting him up and exercising him (p10)

So I work with in health visiting team. I see lots of children, I refer to Physio for a lot of families. I didn't know what physios did (p2)

so I wouldn't even know what physiotherapy would be for her (P12)

the focus in terms of the doctors and his kind of wellness was not anywhere related to his development (P4)

the physiotherapist come out and was moving his legs for him and moving him and turning him to the side. I don’t understand why they did that or what they were doing (p1)

Community physiotherapy provision

The last community Physio we saw wasn't that great. She was a very young Physio and not against young Physios, she just didn't know **** background. I had to explain it from literally dot all the way up (p2)

But yeah it's just frustrating that it was an instant discharge (p7)

It's just like banging your head against a brick wall. It's really, really difficult and it's really frustrating, because all you want is the support for your child (p7)

I think having like a 10-15 min workshop on them isn't enough to be trusted to be able to go and feel confident to do that on your own (p8)

the last physio that saw her said that there's not much that she can do about pain other than just giving her Calpol, but I kinda just can't help but think there must be a reason why her ankle is hurting her all the time (p2)

They did give us and it didn't wasn't the best situation. The gators they didn't. They didn't go down very well. I really didn't like those, and ** (child) hated them, and I just got to a point I was. I'm not forcing these. I'm finding it miserable. She's finding it miserable. It wasn't gain, it wasn't gaining anything (p8)

We saw them at a health clinic to start with for the 1st assessment for the initial one, and they've been out to school once in, what years he in now, he’s going into year 4, so once in 3 years they've been out to school to see him, and that was 2 years ago (p7)

They've been doing it for a year, but they're like nobody's trained us so we can't do it. They employ a Physio on site as a specialist they have an on-site physio. I said can the Physio not show you what to do? No, because it's an NHS program she can't do it (p9)

in my head physios belong in hospital (p3)

I don't know if [physio] knew about congenital heart disease quite as much as we told her now (p6)

Probably waiting a few weeks for appointment, but when they rang me up to book an appointment, he was due to come in and have his surgery, so I told them that, and then they said, Oh, well, usually, if we ain't seen you within a week or 2, we'll take you off the list (p11)

I just want them to reassess him. Whether it's the same as last time, where they give us all the exercises and say, right off you go that's fine. As long as I know that we're doing the right thing by him and giving him the relevant exercises to help build those muscles (p7)

Logistical challenges

so next week the Thursday I have 3 appointments in 3 different hospitals come through for the same morning (P3)

Clashes is the big one at the minute that's come up (p4)
I can't even begin to tell you how many times the appointments are the same, or you know, she's got like about 5 appointments coming up in August (p2)

she didn't want him to have to come out of school all the time to, because obviously we had cardiac appointments (P5)

The volume can be intense at times, um especially if they all suddenly fall together (p7)

But we live in a more rural place now, and if I didn't drive that would be a huge barrier (p10)

I feel like I'm there a lot. I feel like I need a season pass for the parking (p8)
if we didn't have 2 cars for quite a long time, and there are not many buses around where we are. They don't come. They come twice a day, maybe 3 times a day, to *** (location), and then you'd have to catch another bus, which is fine. But that's a that's a whole day.

And hopefully your physio appointment, or any appointment will be within the timeframe you can catch a bus, or you have to get taxi (p6)

You know she's got a blue badge, so I just park and lock up, and you know it's easy (p9)

Family risk assessment

I'm winding myself off about it, and it's over a week's time and there's 2 other appointments in between that. So it's really not important right now (p3)

want more people to be able to help him in the future and not necessarily have to do what I do and just explain everything in detail all the time, because it's an extensive that takes time to explain (p4)

and will just cling on to us, and you won't get anything from her (p8)

Every time we go he does the heart scan, which is just, you know, a little doppler, and all she does is take her top off, puts a bit of gel on, and it just goes, and it doesn't hurt her, but she screams the whole time screams (p12)

he's been diagnosed with like he's got PTSD pretty bad from his hospital stays (p6)

I don't know if that's partly because he goes to hospital all the time. A lot of things are triggered around touch. So if you touch him too much, he gets really angry, and he wants you to get off him (p1)

I think I really suffered. If I, you know, on a personal level. I developed PTSD from this all not surprised. So I've been mentally having to deal with myself. So we've had some non-heart issues, she broke her arm, you know, as kids do, and I was like, I can't go into the hospital (p8)

the state he was is in during his cardiac checkups you wouldn't notice anything physically anyway (p5)

you couldn't get him into just the building complex you know the gates of **** (hospital), going through those gates holding him in your arms, you would feel him tense and like start and he couldn't even, felt quite bad for the nurses like, because he couldn't see someone anyone at all couldn't see anyone in uniform without completely freaking out, crying, screaming, trying to get away and was really tough (p5)

<u>Ideal physiotherapy service</u>

Flexible and personalised service

if you need to contact somebody contact this person, give them an email. So, it could be something really simple (p3)

I think physio can have a place, because I think if he's, you know, if he's behind now, and he's walking, if he still struggles with, you know, one side of him slightly delayed. He's still

going to need help with that. I don't feel like it might even out a little bit, but I don't feel like that support isn't gonna be needed, because it just he's still going to need to keep up strength in those, to even out that side of his body (p4)

we know that all throughout her life she's going to require some kind of physio and some kind of help kind of thing (p9)

Yes, so yeah, it's sort of like I fear that she'll probably always need sort of like some kind of intervention, whether it be physio or whether it be occupational therapy. You know I still think that she will possibly need more. Whether it be small or whether it be big, I'm not too sure (p2)

then they can look at the child, and then it will need to be tailored (p3)

Showing me how to do, not interested in something to read (p11)

I think it's just peace of mind for us that if it's stuff we've got to do that we're showing how to do it properly. But if we've got any questions (p7)

Probably once or twice in his life now it probably wouldn't be often, maybe even just a phone call if I had a physio call me and just say, do you have any questions (p1)

I think, and probably setting up like a dedicated email, or mobile, or something that you could message into. Our specialist nurse is [name] so you could text, you can text her because often I don't need something urgently. (P3)

Standardised service provision

just a nice, easy, accessible one, where, like yearly assessments and reviews can be done or 6 monthly, if that's what children needed (p7)

I think going forwards the physio is gonna continue to be really, really crucial in his development, because if he spends a long time not being mobile again post-surgery, I'm worried he will lose some of those skills (P4)

we're about to go to clinic this week and actually, I would say, that's a perfect time to catch a parent. You know, we're out of the like crisis point, I suppose we're home. He's now being a normal baby, but there's probably a few things that could do with like tidying up (p3)

I fear that she will probably still need physical Physio. I feel like we will still need to be re-referred back into community (p2)

So definitely in terms of the recovery just some information on what it will have done to his muscles, and how to help that kind of strengthen again (p5)

I think actually keep almost trying to run a physio clinic for these cardiac babies so that they can be reviewed (p3)

I think, being able to get a hold of them, or at least having yearly reviews instead of just as, and when school or myself feel fit to get them back in. If that makes sense (p7)

If you're stable and everything, I'd probably say like once every 6 months. It would be sufficient, because, say, you don't want to be or maybe 3 months. I I guess it just depends on the severity of a child and what the needed is (p9)

It would be so good if everybody could talk to everybody and just have go to the hospital one day and then just get seen by everyone (p2)

Once a month, every maybe 6 weeks (p5)

So I think initially, some sort of phone or email access to then work out what that child and family need, and that may be coming into hospital for review or it might be just, oh, actually, Mom, just go and do this, and it's fine (p3)

Physiotherapy link for CHD

I think it's important that they need to know, so that you know what their limits are, and I think it's important to know that actually, this child's plumbing isn't the right way around. So actually, something might be a little bit more exhausting than for another child (P8a)

I think I would feel confident with the people that have looked after him in hospital and have that that specialist knowledge base (p3)
But then I think it's important that they need to know, so that you know what their limits are, and I think it's important to know that actually, this child's plumbing isn't the right way around (p8b)
Freferra
his heart plays a big part in their life, doesn't it? It all depends on where what stage they're at, because he's gonna have to have another operation further down the line so that could always impact on well, it's the oxygen, isn't it? And how it affects the body, and that so I don't think it would ever hurt for everybody to know like what the implications could be (p7)

they understand developmental milestones you know, every professional will have some knowledge of that. But the journey that these parents have been on is not simple (p3)

they understand developmental milestones you know, every professional will have some knowledge of that. But the journey that these parents have been on is not simple (p5)

it's they don't need to know about I think the heart condition to do the physio well, they need to know about the heart condition to win over the parents (p8b)

I think having a point of contact (p7)

I think more checkups or more opportunities to kind of practice or talk through it with someone (p8)
Parental education and support

But as long as I know that there is support there, and that puts my mind at rest (p12)

Maybe so like a leaflet or something, you know. So I don't think I was ever given sort of like a leaflet or anything like you would with cardiac surgery. So just maybe like a little leaflet like included in the pack, like what will happen when your child goes into hospital (p2)

So they know what to look out for, and things like that. Things, if there are certain things they can do to improve on it, and stuff, I think (P11)

it's sort of like being included in the whole information would be really useful, I think, especially if maybe the parents 1st child or they're unaware of, like myself, what Physio actually did and PIC and hospitals are really scary when you’ve not been there (P2)

in terms of having had the open heart surgery. It might have been useful to have a bit more information about what, if any kind of things you can do to make sure that that's all healed and strengthened (p5)

Physio would give me more confidence in what to tell people he can do (p1)

Definitely that would be useful for an antenatal contact before you then get the child in front of you, and it's just a sort of a whirlwind explosion (p3)
