# Supplementary material for: Exploring parental perspectives of physiotherapy in children with congenital heart disease: a qualitative study
Source: BMJ Paediatr Open. 2025 Nov 10;9(1):e003705. doi: 10.1136/bmjpo-2025-003705 (PMC12603715; doi:10.1136/bmjpo-2025-003705)
Supplement: online supplemental file 2 [file bmjpo-9-1-s002.pdf]

UNIVERSITY OF BIRMINGHAM

Birmingham Women's and Children's NHS Foundation Trust

Topic Guide for Parental Interviews

Parental perspectives of physiotherapy in children with congenital heart disease

Introduction

- **Introduce self (provide context to study and interview)**

My name is Steph Clarke, I am a Physiotherapist working at Birmingham Children's Hospital. I am also a research master's student at the University of Birmingham.

- **Thank participants for taking part**

Thank you for agreeing to take part in this interview.

Plan for the interview

- During this interview, we will discuss the following topics: the development of your child, how you feel their development could be supported and the role of physiotherapy in this
- The interview will be audio and video recorded and will last no longer than one hour
- Field notes may be taken
- You can ask questions at any stage of the interview

Study overview

I would like to hear your perspectives on your child's physical development and how you think physiotherapy could help.

Confidentiality and Consent

- The recording, transcription and notes will be kept securely, and the discussion will be confidential.
- All identifiable information will be removed from the transcript. If there are any questions you would prefer not to answer, then please say and you will not have to answer that question.

- You are also welcome to withdraw from the interview at any stage, please do so by ending the Zoom call.
- Please can I check if you have read the participant information sheet? Did you sign the consent form?
- Do you have any further questions?

Please can I confirm you are happy to continue with the interview? And you are happy to be recorded?

START RECORDING

Questions

1. **Parent information**

Could you start by telling me a little about yourself and your child?

Prompts: how old are their child/children? what is their child's diagnosis? what surgery has the child had? What is their family set-up? What other past medical history do they have?

[Ice breaker question. To help the researcher understand the experience of participants]

(Link – Now I have an understanding of you as a family, it would be helpful to explore your child’s development)

2. **Child’s development or function.**

I am interested in understanding your thoughts regarding your child's development- can you tell me about this?

Prompts: what activities does your child like to do? Can they do everything that their friends can do? What difficulties did they or do they face? Have you or do you currently have concerns about how your child has developed or how they move? Do they have any underlying neurological diagnosis/PMH?

[unpick what concerns they had/have with their child’s motor function]

(Link –Now we have discussed your child's development it would be good to explore more about physiotherapy)

3. **Accessing Physiotherapy**

Could you tell me about any experiences your child has had with physiotherapy?

Prompts: Why have you accessed physiotherapy? Where was physiotherapy provided? How often were you seen? How were you referred? What is your understanding of physiotherapy? Did you feel that it was beneficial?

[unpick who is involved with physiotherapy, what service is offered, what do families understand of physiotherapy]

(Link – Let's move on to think about how your child's development could be supported)

4. **Physiotherapy role**

I am interested in understanding from your perspective how you feel your child's development could be best supported by a physiotherapist and what could have improved your experience of physiotherapy- can you tell me a bit about this?

Prompts: When should support be offered? how often should support be offered? where should support be offered? who should provide the support? What do they think would have helped your child at the time you were worried about them? What would you have liked in an ideal world? What support would you like in the future?

[Aim to explore how a physiotherapy intervention would look]

(Link – the final area I want to explore is accessing services)

5. **Accessing Physiotherapy**

What would impact you as a parent being able to access/attend physiotherapy for your child?

Prompts: what would stop you from attending an appointment? What would make it difficult for you to access physiotherapy? What would make it easier for you to attend an

appointment? What makes it difficult to attend appointments? How have you overcome these difficulties?

[Aim to understand barriers and facilitators to services]

Of the questions I have asked today is there anything else you would like to discuss?

Thank you for participating in today's interview. It has been very insightful and helpful. I want to remind you that the conversation we had today is confidential and any comments featured in the study will be anonymised. If you have any concerns or complaints, please refer to the participant information sheet for information on whom to contact.
