# Supplementary material for: Exploring parental perspectives of physiotherapy in children with congenital heart disease: a qualitative study
Source: BMJ Paediatr Open. 2025 Nov 10;9(1):e003705. doi: 10.1136/bmjpo-2025-003705 (PMC12603715; doi:10.1136/bmjpo-2025-003705)
Supplement: Supplementary file 3 [file bmjpo-2025-003705.reviewer_comments.pdf]

PEER REVIEW HISTORY

BMJ Paediatrics Open publishes all reviews undertaken for accepted manuscripts. Reviewers are asked to complete a checklist review form and are provided with free text boxes to elaborate on their assessment. These free text comments are reproduced below.

ARTICLE DETAILS

**TITLE (PROVISIONAL)**	Exploring parental perspectives of physiotherapy in children with congenital heart disease: a qualitative study
**AUTHORS**	clarke, stephanie; Menzies, Julie; Shkurka, Emma; Drury, Nigel

VERSION 1 - REVIEW

**REVIEWER NAME**	Bethan Holmes
**REVIEWER AFFILIATION**	University of Birmingham, Department of Nursing and Midwifery
**REVIEWER CONFLICT OF INTEREST**	N/A
**AI DISCLOSURE**	No
**DATE REVIEW RETURNED**	03-Jul-2025

**GENERAL COMMENTS**	Your paper offers valuable insights into how parents understand their child’s development following CHD surgery and how this shapes their engagement with physiotherapy services. The discussion effectively connects the findings to existing literature and highlights important implications for service improvement. One small omission is the completed COREQ checklist in the supplementary material; including this would strengthen the study’s transparency and enhance its methodological rigour.

**REVIEWER NAME**	Jane Hopkins
**REVIEWER AFFILIATION**	Torres and Cape Hospital and Health Service
**REVIEWER CONFLICT OF INTEREST**	N/A
**AI DISCLOSURE**	No
**DATE REVIEW RETURNED**	04-Aug-2025

**GENERAL COMMENTS**	Having parents provide their ideas for ideal physiotherapy service provision is a great focus. Will clinical practice guidelines be developed? It would be beneficial to explore/ discuss parental well being and experience of trauma during the uncertain period as that may impact recollection. Also whole of family response, would be good to have more dads as we know experiences can be very different. The mportance of physiotherapist developing a relationship and being family led was clearly demonstrated in discussion. Exploring preferred format of education for families would also be useful information.
